# The potential therapeutic effect of phosphodiesterase 5 inhibitors in the acute ischemic stroke (AIS)

**DOI:** 10.1007/s11010-023-04793-1

**Published:** 2023-07-03

**Authors:** Raed AlRuwaili, Hayder M. Al-kuraishy, Mubarak Alruwaili, Amira Karam Khalifa, Athanasios Alexiou, Marios Papadakis, Hebatallah M. Saad, Gaber El-Saber Batiha

**Affiliations:** 1 Department of Internal Medicine, College of Medicine, Jouf University, Sakaka, Saudi Arabia; 2 Department of Clinical Pharmacology and Medicine, College of Medicine, ALmustansiriyia University, Baghdad, Iraq; 3 Department of Medical Pharmacology, Kasr El-Ainy School of Medicine, Cairo University, El Manial, Cairo, 11562 Egypt; 4 Lecturer of Medical Pharmacology, Nahda Faculty of Medicine, Beni Suef, Egypt; 5 Department of Science and Engineering, Novel Global Community Educational Foundation, Hebersham, NSW 2770 Australia; 6 AFNP Med, 1030 Vienna, Austria; 7 Department of Surgery II, University Hospital Witten-Herdecke, University of Witten-Herdecke, Heusnerstrasse 40, 42283 Wuppertal, Germany; 8 Department of Pathology, Faculty of Veterinary Medicine, Matrouh University, Marsa Matrouh, 51744 Egypt; 9 Department of Pharmacology and Therapeutics, Faculty of Veterinary Medicine, Damanhour University, Damanhour, 22511 AlBeheira Egypt

**Keywords:** Acute ischemic stroke, PDE5 inhibitors, Neuroinflammation, Inflammatory signaling pathways

## Abstract

Acute ischemic stroke (AIS) is a focal neurological disorder that accounts for 85% of all stroke types, due to occlusion of cerebral arteries by thrombosis and emboli. AIS is also developed due to cerebral hemodynamic abnormality. AIS is associated with the development of neuroinflammation which increases the severity of AIS. Phosphodiesterase enzyme (PDEs) inhibitors have neuro-restorative and neuroprotective effects against the development of AIS through modulation of the cerebral cyclic adenosine monophosphate (cAMP)/cyclic guanosine monophosphate (cGMP)/nitric oxide (NO) pathway. PDE5 inhibitors through mitigation of neuroinflammation may decrease the risk of long-term AIS-induced complications. PDE5 inhibitors may affect the hemodynamic properties and coagulation pathway which are associated with thrombotic complications in AIS. PDE5 inhibitors reduce activation of the pro-coagulant pathway and improve the microcirculatory level in patients with hemodynamic disturbances in AIS. PDE5 inhibitors mainly tadalafil and sildenafil improve clinical outcomes in AIS patients through the regulation of cerebral perfusion and cerebral blood flow (CBF). PDE5 inhibitors reduced thrombomodulin, P-selectin, and tissue plasminogen activator. Herein, PDE5 inhibitors may reduce activation of the pro-coagulant pathway and improve the microcirculatory level in patients with hemodynamic disturbances in AIS. In conclusion, PDE5 inhibitors may have potential roles in the management of AIS through modulation of CBF, cAMP/cGMP/NO pathway, neuroinflammation, and inflammatory signaling pathways. Preclinical and clinical studies are recommended in this regard.

**Graphical abstract:**

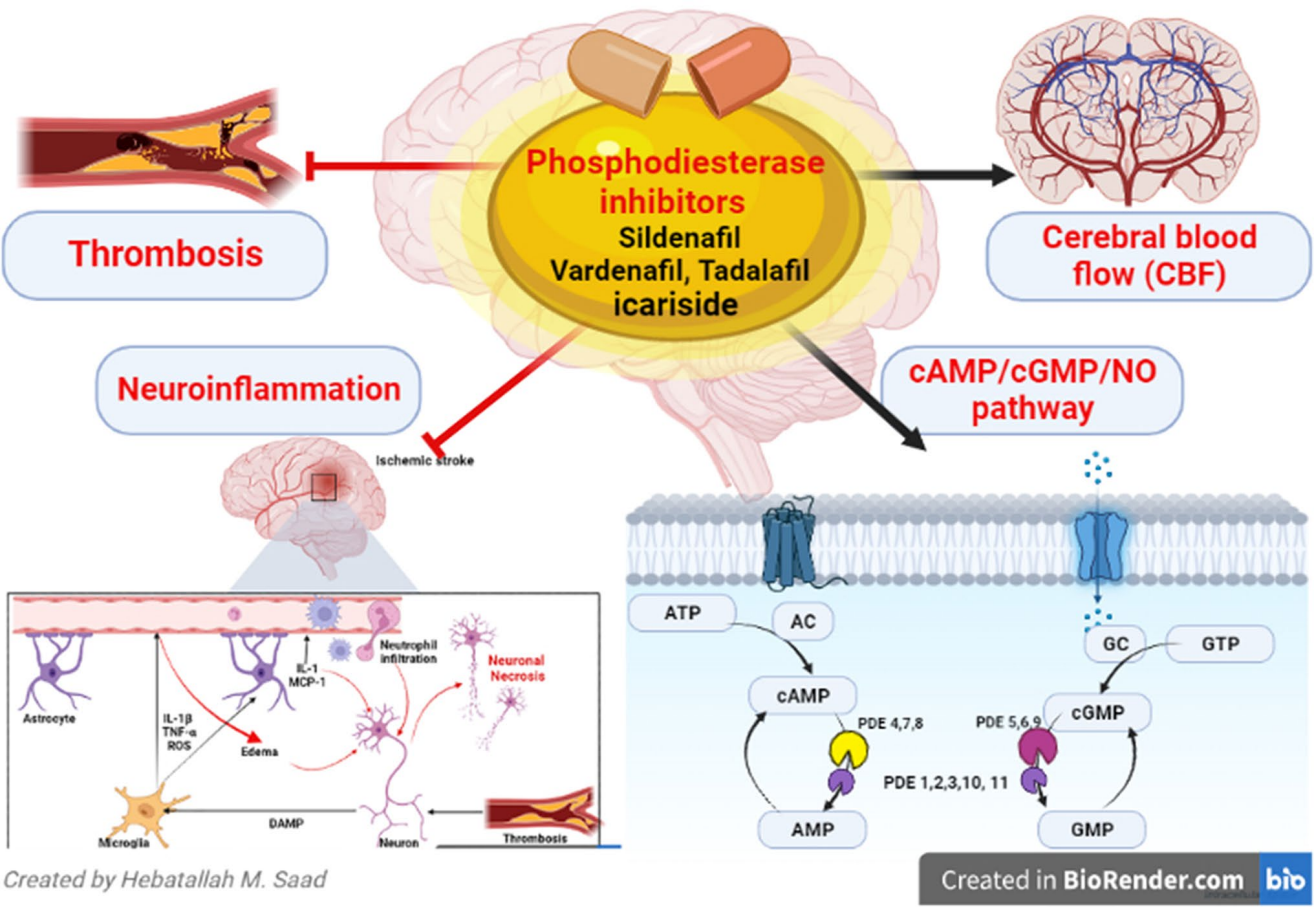

## Introduction

**Fig. 1:**
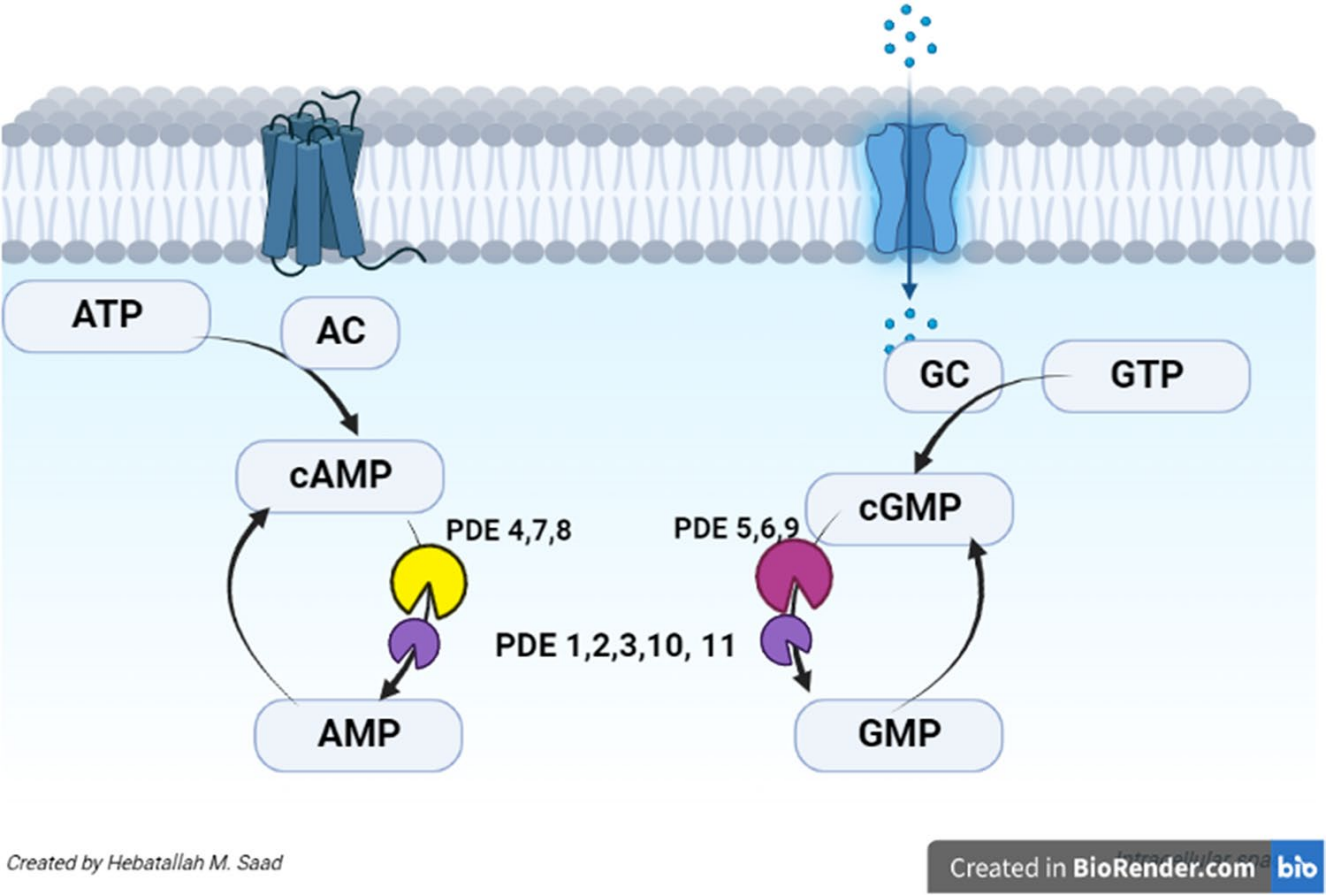
Role of phosphodiesterase enzymes (PDEs) in the metabolism of cyclic guanosine monophosphate (cGMP) and cyclic adenosine monophosphate (cAMP). cGMP is synthesized from guanosine triphosphate (GTP) by the action of guanylate cyclases (GC) and then hydrolyzed by the PDE 5, 6 and 9 to GMP. cAMP is synthesized from adenosine triphosphate (ATP) by the action of Adenylate Cyclases (AC) and then hydrolyzed by the PDE 4, 7 and 8 to adenosine monophosphate (AMP). Additionally, PDE 1, 2, 3, 10 and 11 hydrolyze both cAMP and cGMP

Phosphodiesterase enzymes (PDEs) are class of enzymes involved in the hydrolysis of cyclic guanosine monophosphate (cGMP) and cyclic adenosine monophosphate (cAMP) [1]. There are 11 PDE families, of note, PDE 1, 2, 3, 10 and 11 hydrolyze both cAMP and cGMP, while PDE 4, 7, and 8 hydrolyze only cAMP, and PDE 5, 6 and 9 hydrolyze only cGMP [2, 3]. Recent PDE12 types hydrolyze 2–5-oligoadenylate which regulates immune response (Fig. 1) [4].

In early 1970, Weiss and colleagues identified the PDE isolated from rat brains. Later on, selective inhibitors of PDE were recognized by the same researchers [5]. Like other PDEs, PDE5 contains 2 subunits 100 kd in size which has a cGMP binding site and a catalytic domain. The cGMP binding site is upregulated by the action of protein kinase c (PKC). Three types of PDE5 are present including PDE5A1, PDE5A2 and PDE5A3 with subtypes from 1 to 3. In general, PDE5 is highly expressed in most tissues mainly in the cardiovascular system [6, 7]. Growing evidence indicates that cGMP plays an important role in neural development and neurotransmission. PDE5 mRNA was abundant in cerebellar Purkinje cells, with minimal, detected in the olfactory bulb, cortical layers, and in hippocampus. Thus, it appears that differential expression of PDE isoforms may provide a mechanism to match cGMP hydrolysis to the functional demands of individual brain regions [8]. Moreover, PDE5 is highly expressed in microglia and astrocytes that contribute to the development and progression of neuroinflammation [9].

**Fig. 2:**
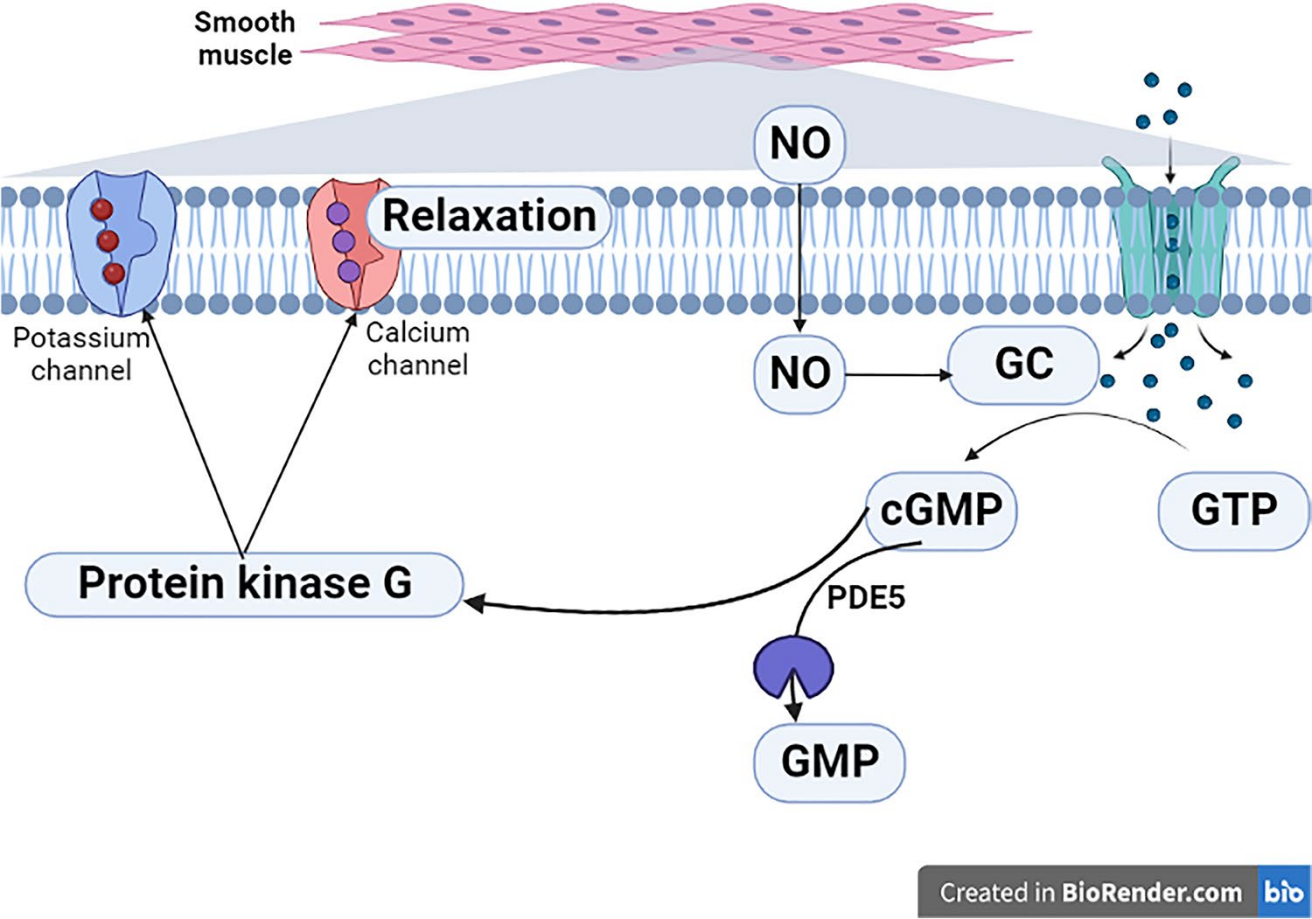
Phosphodiesterase enzymes (PDEs) and nitric oxide (NO). NO penetrates smooth muscle cells and binds to guanylate cyclases (GC) resulting in the formation of cyclic guanosine monophosphate (cGMP) from guanosine triphosphate (GTP). cGMP activates cGMP-dependent protein kinase leading to a decrease in intracellular calcium and relaxation of arterial smooth muscle subsequently

Furthermore, PDE5 inhibitors like sildenafil, vardenafil and tadalafil are the main drugs in this class. However, icariin and zaprinast have weak effects on PDE5. Despite PDE5 inhibitors having the same mechanism; however, the onset and duration of PDE5 that affect the efficacy and adverse effects of these drugs are different [10]. For example, a short-duration PDE5 inhibitor sildenafil blocks PDE6 which is present in the retina causing reversible color vision disorders [11] while a long-duration PDE5 inhibitor tadalafil also blocks PDE11 which is found in the prostate that may affect fertility [12]. The most adverse effects associated with the use of PDE5 inhibitors are headache, dyspepsia, nasal congestion, flushing and hearing loss [13]. Uses of PDE5 inhibitors during pregnancy increase the risk for the development of fetal growth restriction and optic neuropathy [14]. Concomitant use of PDE5 inhibitors with other vasodilator agents and nitrate should be omitted within 24–48 h due to the risk of severe hypotension and shock (Fig. 2) [15].

PDE5 inhibitors prolong the action of cGMP by inhibiting its degradation by the enzyme PDE5, which is found throughout the body. In the penis, PDE5 inhibitors potentiate the effects of cGMP to prolong erections and increase sexual satisfaction. However, PDE5 inhibitors do not cause erections without sexual stimulation [16]. As well as their hemodynamic effects, PDE5 inhibitors have also been shown to have anti-inflammatory, antioxidant, antiproliferative, and metabolic properties in several experiments [17].

PDE5 inhibitors are mainly indicated in the management of erectile dysfunction, pulmonary hypertension and heart failure [15, 18]. Recent research proposed that PDE5 inhibitors could be effective in treating diabetes mellitus [19], chronic kidney diseases [20], intermittent claudication [21] and myocardial infarction [22]. In addition, PDE5 inhibitors could be effective in the management of acute ischemic stroke (AIS) [23]. Thus, the present study aimed to elucidate the mechanistic role of PDE5 inhibitors in the management of AIS.

## Acute ischemic stroke

**Fig. 3:**
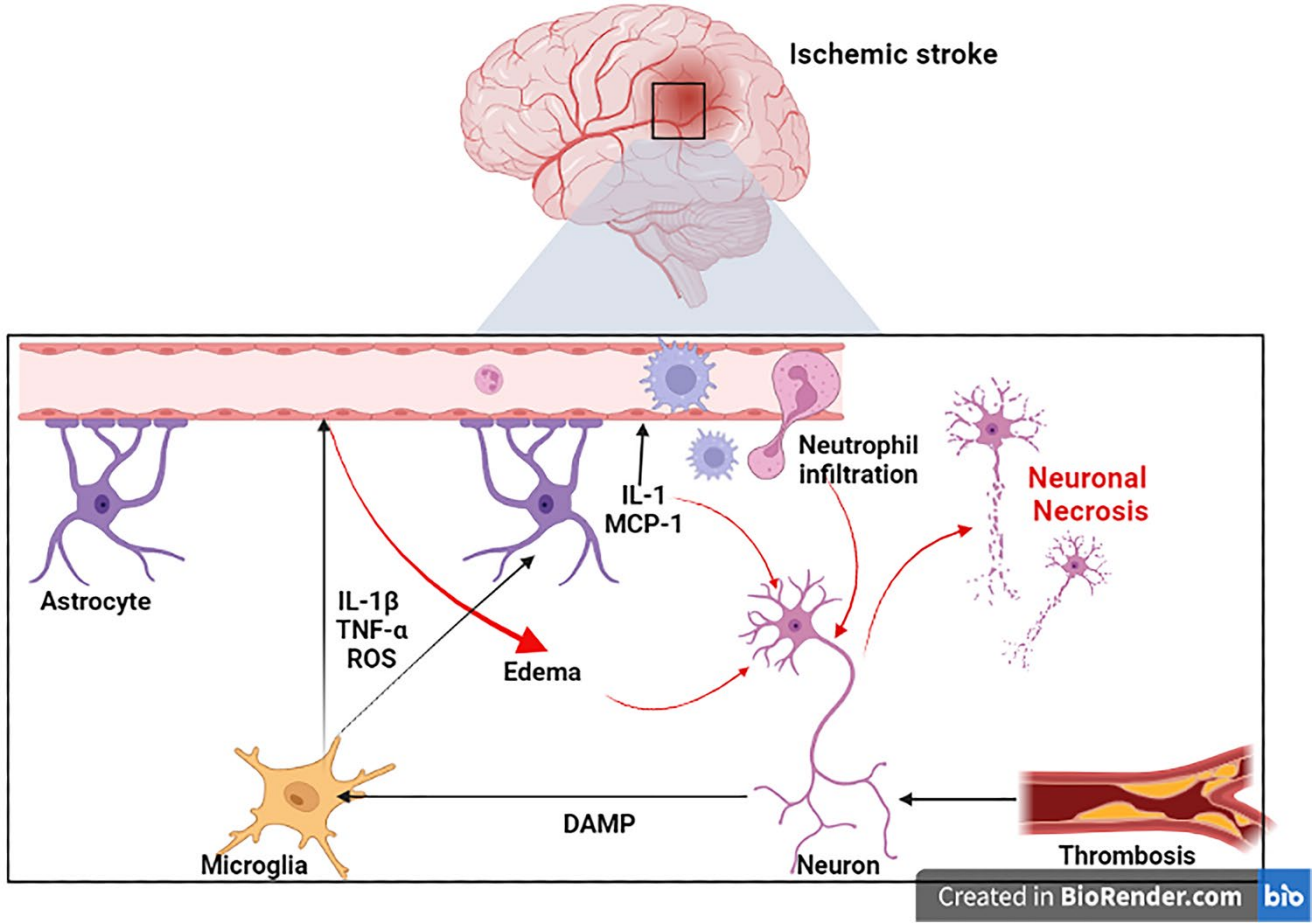
Mechanism of neural death following reduction of cerebral blood flow in ischemic stroke. During ischemic stroke, damage-associated molecular pattern (DAMP) is released from injured neurons which activates microglia to release various pro-inflammatory cytokines like interleukin (IL)-1β and tumor necrosis factor-alpha (TNF-α) in addition to reactive oxygen species (ROS) leading to weakness of blood–brain barrier with the subsequent lifting of astrocytic end feet from the basement membrane, cerebral edema and leukocytic infiltration to cerebral parenchyma, where they release proinflammatory mediators as IL-1 and monocyte chemotactic protein 1 (MCP-1) that exacerbate neuroinflammation

AIS is a focal neurological disorder that accounts for 85% of all stroke types, due to occlusion of cerebral arteries by thrombosis and emboli. AIS is also developed due to cerebral hemodynamic abnormality [24]. AIS is the leading cause of mortality in developed countries it represents about 11.9% of death annually [24]. Different risk factors contribute to the development of AIS including modifiable risk factors such as hypertension, diabetes mellitus, obesity, and cigarette smoking, and non-modifiable risk factors such as male gender, old age, low birth weight at birth and ethnicity [25, 26]. AIS is mainly developed due to rupture of atherosclerotic plaque with subsequent thrombosis in the cerebral vessels resulting in brain infarction, ischemic-reperfusion injury and development of peri-infarct inflammation and complicated neuroinflammation [27]. Progressive neuroinflammation associated with AIS results in neuronal injury and axonal degeneration. These changes induce the generation of reactive oxygen species (ROS) which trigger blood–brain barrier (BBB) dysfunction, Ca neuronal overload and excitotoxicity. During the development of AIS, activated microglia release various pro-inflammatory cytokines like interleukin (IL)-1β and tumor necrosis factor-alpha (TNF-α). As well, damage-associated molecular pattern (DAMP) which is released from injured neurons activates toll-like receptor 4 (TLR4) and nuclear factor kappa B (NF-κB) with more release of pro-inflammatory cytokines and development of neuroinflammation (Fig. 3) [28].

These findings suggest that AIS and associated neuroinflammation lead to progressive neuronal injury due to disruption of BBB integrity and propagation of excitotoxicity and oxidative stress. Targeting neuroinflammation with anti-inflammatory and antioxidant agents may limit AIS complications.

## PDE5 inhibitors and acute ischemic stroke

It has been shown that administration of PDE5 inhibitors in patients with high cardiovascular risk have been shown to ameliorate endothelial function parameters, reduce plasma concentrations of endothelin 1 (ET-1) and endothelial inflammatory mediators and increase levels of circulating endothelial progenitor cells [29]. Favorably, several studies have indicated that PDE5I-mediated improvements in endothelial function may be sustained for weeks to months after discontinuation of long-term treatment [29]. Pauls et al. [30] study highlights the potential for therapeutic intervention in both ischaemic stroke and neurodegenerative disorders. However, the few studies investigating these effects in humans are limited by their focus on cerebral blood flow. Endothelial dysfunction is a hallmark of cerebrovascular disease, including AIS. Modulating endothelial signaling by cAMP and cGMP is a potential therapeutic target in stroke. Inhibitors of the PDE enzymes may restore cerebral endothelial function [31]. PDE5 inhibitors promote angiogenesis, microcirculation and improved functional recovery; all are important in stroke prevention and recovery, and effects should be further evaluated in humans [31]. Pooled data regarding myocardial infarction and cardiovascular death from more than 120 clinical trials of sildenafil citrate conducted from 1993 to 2001 showed that the rates of Ml and cardiovascular death were low and comparable between men treated with sildenafil and those treated with placebo [32]. In tadalafil clinical trials, the incidence of cardiovascular adverse events in patients receiving tadalafil was low and comparable to placebo. Tadalafil did not increase the rate of cardiovascular mortality compared with reported rates from epidemiological studies [33]. This favorable cardiovascular safety profile for tadalafil is important because men with erectile dysfunction commonly have cardiovascular disease and may seek medical therapy for erectile dysfunction. In a double-blind, placebo-controlled, cross-over trial, participants received tadalafil (20 mg) and placebo on two visits ≥ 7 days apart illustrated a non-significant effect of single-administration tadalafil on CBF [34].

### PDE5 inhibitors and cerebral blood flow

It has been reported that PDE5 inhibitors improve CBF through the modulation of cerebral cAMP/cGMP [35]. An experimental study illustrated that administration of PDE5 inhibitor sildenafil 20 mg/kg reduces infarct size, neuronal loss and pathological electroencephalogram (EEG) changes in rats with mild AIS [36]. Silver et al. [37] found that treatment of AIS patients with sildenafil 25 mg/kg for 2 weeks improved clinical outcomes. However, different clinical case reports illustrated that the use of PDE5 inhibitors may trigger the propagation of AIS [38, 39]. Morgan et al. [38] illustrated that a 50-year-old man develop right hemiparesis following ingestion of 50 mg of sildenafil. Likewise, treatment with sildenafil or tadalafil may increase the risk of AIS in young adults [39]. Though, most large-scale clinical studies did not confirm the association between the use of PDE5 inhibitors and the risk of AIS [40]. PDE5 is highly expressed in meningeal and cerebral vessels [41]. An elegant experimental study illustrated administration of sildenafil at 10 mg/kg /day for 1 week improved CBF in rats [42]. Decreased regional CBF is the principal factor determining the topography of tissue injury after hypoxic-ischemic brain injury in the immature rodent brain [43]. Therefore, boosting NO-cGMP signaling through PDE-5 inhibition with sildenafil would improve microcirculatory CBF, and reduce ipsilateral hypoxic-ischemic brain injury and associated neuroinflammation and motor deficit [43]. It has been shown that sildenafil attenuates neonatal hypoxic-ischemic brain injury in rats by increasing and redistribution of CBF [44]. Sildenafil at a dose of 10 mg/kg was able to increase CBF to levels similar to those measured in normal animals, strongly supporting the occurrence of pharmacological vasodilation and collateral recruitment/patency through the NO-cGMP pathway [45]. Conversely, inducible nitric oxide (iNO) at 10 or 40 ppm has been reported to increase lesion size when given after the hypoxic-ischemic brain injury [46]. Together, these data suggest that NO-dosage and time period of exposure are crucial and vary depending on the vascular responses specific to each model of ischemic and hypoxic-ischemic brain injury. Thus, PDE5 inhibitors may selectively improve CBF with subsequent improving the outcomes of AIS patients though NO-dependent mechanism.

Moreover, tadalafil may affect regional CBF after a single dose and continuous dose in AIS patients [47]. A prospective study involved 30 males with erectile dysfunction and previous AIS showed that administration of tadalafil reduced CBF in the area adjacent to the infarcted area compared to the baseline as evaluated by single-photon emission computed tomography [47]. This verdict suggests a differential effect of tadalafil and sildenafil on CBF in AIS patients. Nevertheless, tadalafil improves clinical outcomes and neurological functional recovery in rats with experimental AIS [48]. Tadalafil promotes cerebral vascular density around the brain ischemic region via increasing neuronal cGMP with subsequent enhancement of neurogenesis and angiogenesis [48]. A recent pilot study demonstrated that the administration of tadalafil promotes perfusion in AIS patients [49]. Treatment with tadalafil 20 mg in two separate doses compared to placebo in 20 AIS patients showed that tadalafil increases cortical microvascular oxygen saturation via improvement of cerebral perfusion [49].

Changes in CBF following the administration of PDE5 inhibitors have been reported in older people with brain disease, though the results were quite diverse [50]. In older male subjects with a history of ischemic stroke, a mosaic of changes in regional CBF followed tadalafil treatment [47]. Sildenafil also gave a mosaic of regional CBF changes in men with erectile dysfunction. In subjects with mild cognitive impairment (MCI), diagnosed clinically as an early Alzheimer's disease (AD), there was a small (8%) elevation of global CBF following acute administration of sildenafil (50 mg) [50]. All these studies lacked a placebo-treated control group; hence small PDE5i-dependent effects cannot be distinguished from confounding factors, such as diurnal CBF changes [51]. Healthy young adults showed no change in CBF following acute PDE5i treatment. In young adult male patients with Becker muscular dystrophy, 4 weeks of sildenafil treatment produced a small increase in cerebrovascular reactivity (1.6%) though CBF did not change significantly [52]. These observations proposed that PDE5 inhibitors mainly tadalafil and sildenafil improve clinical outcomes in AIS patients through the regulation of cerebral perfusion and CBF.

### PDE5 inhibitors and brain nitric oxide

Nitric oxide (NO) plays a critical role in the regulation of neuronal differentiation and proliferation in the developing brain [53]. NO regulates CBF under normal physiological conditions, however at the time of ischemia NO level is reduced substantially mainly in the ischemic penumbra, and later at the time of reperfusion, NO increased by about 50% [53]. Different experimental studies observed that NO derived from inducible NOS was neurotoxic in the AIS. Notably, iNOS-positive neurons were increased substantially in the peri-infarct area within three days following induction of focal ischemia [54, 55]. Thus, augmentation of neuronal endothelial NOS by NO donors like L-arginine may produce a neuroprotective effect. L-arginine improves clinical outcomes after AIS through the modulation of neuronal NO [56]. Molnar et al. [57] observed that AIS patients had high levels of L-arginine metabolites due to the development of adaptive mechanisms against endothelial dysfunction in AIS. Various studies revealed that PDE5 inhibitors promote cerebral blood flow (CBF) through the improvement of cerebral NO. Inhibition production of NO by NO synthase inhibitors decreases hypothalamic and cortical CBF [58]. PDE5 inhibitors through increasing cAMP/cGMP improve the biological effects of NO with subsequent improvement of CBF. AIS initiates a cascade of detrimental events including glutamate-associated excitotoxicity, intracellular calcium accumulation, formation of ROS and membrane lipid degradation and/or DNA damage, which lead to the disruption of cellular homeostasis and structural damage in AIS [59]. Indeed, AIS triggers acute inflammation, which exacerbates primary brain damage. Consequently, reducing oxidative stress and downregulating the inflammatory response are options that merit consideration as potential therapeutic targets in AIS [59]. Consequently, NO donors could be neuroprotective agents against the pathophysiology of AIS. Given their short therapeutic window, NO donors seem to be suitable for use during neurosurgical procedures involving transient arterial occlusions, or in the very early treatment of AIS, and also possibly as a complementary treatment for neurodegenerative diseases such as Parkinson's disease (PD) or AD, where oxidative stress is an important promoter of damage [59]. These findings proposed that PDE5 inhibitors improve cognitive function and clinical outcomes in AIS via regulation of neuronal cGMP/NO pathway.

### PDE5 inhibitors and neuroprotection

Several studies have reported that PDE5 inhibitors have a neuroprotective effect through elevating cGMP brain levels. Sildenafil has been found to reduce the neurologic deficit, improve neurogenesis and memory, and promote functional recovery after a stroke and focal cerebral ischemia in young and aged rats [48, 60]. Tadalafil also improved neurogenesis in an embolic stroke model in rats [48]. It has been reported that PDE5 inhibitors inhibit the over-activation of microglia and astrocytes as well as restored neurogenesis [61]. A previous experimental study demonstrated that sildenafil increases brain levels of cGMP, evokes neurogenesis, and reduces neurological deficits when given to rats 2 or 24 h after stroke [60]. A recent experimental study revealed that tadalafil significantly reduced neuroinflammation and promoted neuroprotection and plasticity, regulated the expression of hippocampal glutamate receptors, and restored spatial learning ability and memory through induction of neurogenesis [62]. These data suggest that this drug that is presently in the clinic for sexual dysfunction may have a role in promoting recovery from stroke.

Another study demonstrated that pretreatment with tadalafil attenuated the deleterious effect of cerebral ischemia–reperfusion on infarct size, nitrosative and oxidative stress, memory, and motor coordination [48, 60]. Of note, PDE inhibitors such as dipyridamole and cilostazol have neuroprotective effects via the eNOS-dependent pathway in AIS [63, 64]. In this state, PDE5 inhibitors may reduce neuronal injury in AIS through a NO-dependent pathway. PDE5 inhibitors promote CBF and neurogenesis through augmentation of the NO pathway [58]. As well, sildenafil attenuates pentylenetetrazole-induced convulsion through the regulation of neuronal NO in rats [65]. Devan and coworkers demonstrated that sildenafil improves learning and cognition by increasing neuronal NO in rats [66]. Besides, tadalafil restores short-term memory impairment by inhibiting ischemia-mediated apoptosis of hippocampal neurons [67]. Tadalafil promotes the neuronal cGMP/NO pathway which attenuates the detrimental effects of AIS [67]. Hybrid nitrate and PDE inhibitors improve neuronal activity by regulating cGMP which improves the NO signaling pathway [68]. An experimental study demonstrated that activation of cGMP-dependent protein kinase I improve the outcomes of AIS [69]. Intranasal administration of guanosine advances brain function in AIS in rats [70]. These effects were attenuated by the administration of L-NAME, a nonselective nitric oxide synthase inhibitor [71].

### PDE5 inhibitors and neuroinflammation

It has been shown that AIS is associated with progressive neuroinflammation due to the activation of astrocytes and microglia [72]. AIS-induced neuroinflammation may cause excitotoxicity with subsequent neuronal injury. Supra-physiological concentrations of pro-inflammatory cytokines and ROS during AIS may induce collateral neuronal injury with the reduction of neuronal plasticity [72]. Moreover, activated microglia and associated astrocytes modulate neuroinflammatory reactions and responses, that are protective during the acute phase of ischemic injury, and long-term neuroinflammation may induce progressive neuronal injury [73].

Furthermore, various studies illustrated that PDE5 inhibitors have potential effects on the development of neuroinflammatory reactions in AIS. Peixoto et al. [74] revealed that PDE5 inhibitors attenuate the activation of inflammatory signaling pathways and associated neurodegeneration. PDE5 inhibitors emerged as a potential therapeutic strategy for neuroinflammatory, neurodegenerative, and memory loss diseases [74]. Mechanistically, PDE5 inhibitors produce an anti-inflammatory and neuroprotection effect by increasing expression of NOS and accumulation of cGMP and activating PKG, the signaling pathway of which is thought to play an important role in the development of several neurodegenerative diseases, such as AD, PD, and multiple sclerosis (MS) [74]. The possible use of these drugs in the CNS is related to their ability to cross the BBB. Sildenafil has been described as clearly crossing the BBB and there is also evidence of the ability of vardenafil to do the same [75]. The neuro pharmacokinetic profile of vardenafil after oral administration is detected within 4 min after the dose [76]. However, tadalafil was unable to cross the BBB, though tadalafil reaches the brain in sufficient concentrations to potently inhibit PDE5 [77]. PDE5 inhibitors through augmentation of the cAMP/cGMP/NO pathway regulate platelet activation, immunomodulation, memory consolidation and regulation of neuronal ion channels [74]. Notably, the cAMP/cGMP/NO pathway exerts anti-inflammatory effects via down-regulation expression of endothelial P-selectin with suppression of platelet-leukocyte interactions [78]. As well, increasing intracellular cGMP reduces the production of pro-inflammatory cytokines and the development of oxidative stress [79, 80]. Zhang and colleagues illustrated that PDE5 inhibitors mitigate neuroinflammation, and improve cognitive performance and β-amyloid formation [81]. PDE5 inhibitor icariside inhibits streptozocin-induced cognitive deficits in rats through inhibition activation of NF-κB and release of pro-inflammatory 666 [82, 83]. Besides, PDE5 inhibitors mitigate AIS through the inhibition of neuroinflammation-induced neuronal injury [84, 85]. Moreover, PDE5 inhibitors mitigate inflammatory changes and apoptotic reactions in AIS-induced neuroinflammation through their anti-inflammatory and anti-apoptotic effects. PDE5 inhibitors block the activation of astrocytes and microglia cells with subsequent inhibition release of pro-inflammatory cytokines [40]. As well, PDE5 inhibitors exert anti-apoptotic effects, which attenuate the development of necrosis and synaptic dysfunctions [40]. Therefore, the cAMP/cGMP/NO pathway could be a promising target to mitigate cognitive disorders and neuroinflammation in AIS. PDE5 inhibitors through mitigation of neuroinflammation may decrease the risk of long-term AIS-induced complications. However, administration of PDE5 inhibitors could be detrimental in the acute phase of AIS, and beneficial in the chronic phase of AIS because of a protective effect of neuroinflammation in the acute phase of AIS.

Moreover, many inflammatory signaling pathways are involved in the pathogenesis and propagation of neuroinflammation. For example, Gurgis et al. [86] showed that mitogen-activated protein kinase (MAPK) is engaged in the development of neuroinflammation. Besides, nod-like receptor pyrin 3 (NLRP3) inflammasome is stimulated during brain ischemia causing triggering the development of neuroinflammation [87, 88]. Targeting MAPK, NLRP3 inflammasome and other inflammatory signaling pathways may reduce the propagation of neuroinflammation in AIS. It has been shown that PDE5 inhibitors decrease the severity of pulmonary hypertension by inhibiting NLRP3 inflammasome [89]. In addition, PDE5 inhibitors stimulate angiogenesis by modulating the expression of MAPK [90]. Thus, PDE5 inhibitors through suppression of MAPK and NLRP3 inflammasome may reduce the propagation of neuroinflammation in AIS. However, increasing concentration of NO is associated with the development of neuroinflammation [91]. It is well conventional that glial cells have perilous roles in the inflammatory processes in the CNS. These cells can be activated by a variety of endogenous and exogenous stimuli and can produce high levels of bioactive compounds that are noxious for neuronal cell function. One of the most important molecules released by activated glial cells is the bioactive free radical NO. Though, NO physiologically acts as both a neuromodulator and neurotransmitter in the brain, excess production of NO by glial cells has diverse harmful effects on neuronal function, causing neuronal cell injury/death [92]. The production of NO is induced by overexpression of the iNOS enzyme in glial cells. Besides, iNOS-mediated NO production in neuroinflammatory diseases including MS and AIS are augmented [92]. NO has many homeostatic functions and important roles in inflammation. Within the inflamed brain, microglia and astrocytes produce large amounts of NO during inflammation. Excessive NO causes neuronal toxicity and death, and mesenchymal stem cells can be used as an approach to limit the neuronal damage caused by neuroinflammation [93]. NO produced by the neuronal NOS (nNOS) or iNOS is detrimental in AIS, whereas that derived from the endothelial isoform is beneficial. Though, experimental studies with NOS inhibitors have given conflicting results [94]. CBF was reduced in models of permanent but not transient ischemia when assessed by type of inhibitor; total lesion volume was reduced in permanent models by nNOS and iNOS inhibitors, but not by nonselective inhibitors [94]. A systematic review illustrated that all types of NOS inhibitors reduced infarct volume in transient models. NOS inhibition may have negative effects on CBF but further studies are required. Selective nNOS and iNOS inhibitors are candidate treatments for acute IS [94]. In particular, PDE inhibitors improve the neuroprotective eNO and inhibit harmful iNO during AIS. Taken together, NO and NO donors have conflicting effects on the development of ischemia and neuroinflammation in AIS.

### PDE5 inhibitors and thrombosis

Notably, thrombotic events are associated with the progression and severity of AIS [95]. Released DAMPs during AIS mediate the activation of astrocytes, microglia, and endothelial cells. Also, DAMPs trigger the recruitment and activation of neutrophils with the formation of neutrophil extracellular traps (NETs) [96]. Besides, the expression of high mobility group box 1 (HMGB1) is increased during AIS [95]. Both activated HMGB1 and NETs are involved in the propagation of thrombosis in AIS. These observations proposed that thrombotic events in AIS might be mediated by immunological activation with the progression of immunothrombosis.

It has been reported that PDE5 inhibitors may attenuate the development of thrombosis in AIS. PDE5 inhibitors may inhibit immunothrombosis through suppression of HMGB1and NETs formation. PDE5 inhibitors attenuate inflammatory changes through the inhibition of HMGB1 in diclofenac-induced acute kidney injury [97, 98]. As well, PDE5 inhibitors reduce the propagation of inflammatory disorders via the inhibition of NETs [99, 100]. Therefore, PDE5 inhibitors can prevent the development of immunothrombosis in AIS through modulation of the HMGB1/NETs axis. Sildenafil decreases the risk of thrombosis in AIS [101]. Similarly, sildenafil attenuates the development of pulmonary thrombosis-induced pulmonary hypertension [102]. The anti-thrombotic mechanism of PDE5 inhibitors is related to the inhibition of platelet aggregation through augmentation of the cAMP/cGMP/NO pathway [103]. Yang et al. [104] found that sildenafil decreases the risk of AIS through the inhibition of platelet aggregation and intimal hyperplasia after angioplasty. Of interest, sildenafil blocks platelet activation induced by thrombin and adenosine diphosphate (ADP), mainly through the activation of cGMP-dependent protein kinase (cGK), since cGK inhibitors prevent sildenafil-induced platelet inhibition [104]. Bekels et al. [105] in vivo and ex Vivo studies observed that sildenafil could inhibit ADP and collagen-induced platelet aggregation. In addition, tadalafil mitigates dysfunction of the platelet-endothelial pathway in patients with erectile dysfunction [106].

Furthermore, PDE5 inhibitors may affect the hemodynamic properties and coagulation pathway which are associated with thrombotic complications in AIS. A case–control study evaluated the effects of tadalafil and sildenafil on the thrombotic markers in patients with congenital heart diseases and showed that PDE5 inhibitors reduced thrombomodulin, P-selectin, and tissue plasminogen activator (tPA) [107, 108]. Herein, PDE5 inhibitors may reduce activation of the pro-coagulant pathway and improve the microcirculatory level in patients with hemodynamic disturbances. These verdicts suggest that PDE5 inhibitors can inhibit thrombotic events in AIS through inhibition of platelet aggregation, modulation of the coagulation pathway, and development of immunothrombosis in AIS.

Taken together, the present review highlighted that PDE5 inhibitors have a potential role in the prevention and treatment of AIS through modulation of CBF, cAMP/cGMP/NO pathway, neuroinflammation, and inflammatory signaling pathways. However, the present review had several limitations including the scarcity of clinical studies concerning the role of PDE5 inhibitors in the management of AIS, and the long-term follow-up of AIS patients on PDE5 inhibitors were poorly estimated. Nevertheless, the present review gave a clue that PDE5 inhibitors play a critical role in the mitigation of AIS through different pathways which need to be more estimated in preclinical and clinical studies.

## Conclusions

AIS is a focal neurological disorder that is mainly caused by thrombosis of cerebral arteries. AIS is associated with ischemic-reperfusion injury, peri-infarct inflammation and neuroinflammation. PDE5 inhibitors which are mainly indicated in the management of erectile dysfunction, pulmonary hypertension, and heart failure could be effective in the management of AIS. PDE5 inhibitors mainly tadalafil and sildenafil improve clinical outcomes in AIS patients by regulating cerebral perfusion and CBF via regulation of the neuronal cGMP/NO pathway which also improves cognitive function and mitigate neuroinflammation in AIS. PDE5 inhibitors through mitigation of neuroinflammation may decrease the risk of long-term AIS-induced complications. PDE5 inhibitors can inhibit thrombotic events in AIS through inhibition of platelet aggregation, modulation of the coagulation pathway and development of immunothrombosis in AIS.

Taken together, the present review highlighted that PDE5 inhibitors have a possible role in the prevention and treatment of AIS through modulation of CBF, cAMP/cGMP/NO pathway, neuroinflammation, and inflammatory signaling pathways. The present review gave a clue that PDE5 inhibitors play a significant role in the mitigation of AIS via diverse pathways which need to be more estimated in preclinical and clinical studies.

## Data Availability

Not applicable.
